# Pharmacodynamic Comparison of Ceftolozane/Tazobactam and Ceftazidime/Avibactam, Administered by Intermittent or Continuous Infusion, Against a Clinical Isolate of Carbapenem-Resistant *Pseudomonas aeruginosa* Producing GES *β*-Lactamase in a Hollow Fiber Infection Model

**DOI:** 10.3390/pharmaceutics18040460

**Published:** 2026-04-09

**Authors:** Tae Kun Ahn, Won Gun Kwack, So Young Im, Seo Hyeon Moon, Seok Jun Park, Ki-Ho Park, Eun Kyoung Chung

**Affiliations:** 1 Department of Regulatory Science, Graduate School, Kyung Hee University, Seoul 02447, Republic of Korea; ahnt01@khu.ac.kr (T.K.A.); seohyeon1219@khu.ac.kr (S.H.M.); psjqkr0824@khu.ac.kr (S.J.P.); 2 Department of Pharmacy, College of Pharmacy, Kyung Hee University, Seoul 02447, Republic of Korea; thdudim@khu.ac.kr; 3 Division of Pulmonary, Allergy and Critical Care Medicine, Kyung Hee University Hospital, Seoul 02447, Republic of Korea; wongunnim@khu.ac.kr; 4 Department of Infectious Disease, Kyung Hee University School of Medicine, Seoul 02447, Republic of Korea; 5 Department of Pharmacy, Kyung Hee University Hospital at Gangdong, Seoul 05278, Republic of Korea; 6 Institute of Regulatory Innovation Through Science (IRIS), Kyung Hee University, Seoul 02447, Republic of Korea; 7 Institute of Integrated Pharmaceutical Science, College of Pharmacy, Kyung Hee University, Seoul 02447, Republic of Korea

**Keywords:** carbapenem-resistant *Pseudomonas aeruginosa*, hollow fiber infection model, pharmacokinetics/pharmacodynamics, ceftolozane/tazobactam, ceftazidime/avibactam, infusion method

## Abstract

**Background/Objectives**: Ceftolozane/tazobactam (C/T) and ceftazidime/avibactam (CZA) are critical therapeutic options for multidrug-resistant Gram-negative infections; however, their comparative pharmacodynamics against carbapenem-resistant *Pseudomonas aeruginosa* (CRPA) remain incompletely defined. This study aimed to compare the bactericidal activity of C/T and CZA administered by intermittent infusion (II) or continuous infusion (CI) using a hollow fiber infection model (HFIM) against a clinical isolate of CRPA. **Methods**: Clinically relevant concentration–time profiles for C/T and CZA based on prescribing information were simulated in the HFIM. The standard *P. aeruginosa* strain ATCC 27853 and a GES-producing clinical CRPA isolate were utilized. The primary endpoint was bactericidal activity (≥3 log_10_ CFU/mL reduction from baseline), while secondary endpoints included regrowth prevention and resistance development based on population analysis profiles (PAPs). **Results**: Against the standard strain, both agents achieved rapid killing without regrowth. However, for the GES-producing clinical isolate, C/T failed to achieve bactericidal activity. In contrast, CZA demonstrated sustained bacterial killing activity with the most pronounced early-phase bactericidal activity with CI of CZA (−4.25 log_10_ CFU/mL at 24 h). The bactericidal activity was persistent over 7 days without bacterial regrowth after treatment discontinuation. Conversely, bacterial regrowth occurred with II of CZA after drug withdrawal. PAPs showed the lack of resistance development against CZA, whereas resistance to C/T developed within 48 h after initiating therapy. **Conclusions**: In this HFIM study, CI of CZA demonstrated the most sustained suppression of bacterial growth and prevented resistance emergence against the tested clinical isolate of CRPA producing GES *β*-lactamases. Future clinical studies are warranted to assess the effectiveness of the CI regimen.

## 1. Introduction

The emergence of multidrug-resistant (MDR) pathogens poses an urgent challenge as a worldwide threat to public health; among MDR organisms, carbapenem-resistant *Pseudomonas aeruginosa* (CRPA) represents one of the most concerning isolates [1]. Infections caused by CRPA are particularly problematic because these pathogens can render several classes of antibiotics including carbapenems ineffective, ultimately leading to resistance and extremely limited therapeutic options. Consequently, international recommendations and guidelines, including those from the Infectious Diseases Society of America (IDSA) and the World Health Organization (WHO), have emphasized the urgent need for antibiotics with enhanced activity against MDR pathogens [2,3].

Developing a novel antibiotic agent to target an innovative mechanism with an expanded spectrum of activity against MDR pathogens typically requires substantial resources and time [4]. As a result, rather than developing a first-in-class novel antibacterial agent, alternative strategies have become a mainstay to overcome resistance by targeting the mechanisms of resistance, such as developing β-lactamase inhibitors against an extended spectrum of β-lactamases or carbapenemases [4]. Ultimately, these efforts to improve existing antibiotics by combining them with novel β-lactamase inhibitors led to the successful development of new β-lactam/β-lactamase inhibitor combination products, represented by ceftolozane/tazobactam (C/T) and ceftazidime/avibactam (CZA), which exhibit expanded antibacterial activity against various MDR Gram-negative organisms [5].

Another strategy proposed as a countermeasure against bacterial resistance is to employ alternative administration methods, particularly prolonged infusion (PI) or continuous infusion (CI) of β-lactam antimicrobial agents [6]. The bactericidal activity of β-lactam antibiotics is best predicted by the length of time the unbound drug concentration is maintained above the minimum inhibitory concentration (MIC) over the dosing interval (fT > MIC) [7]. Thus, to account for substantial pharmacokinetic variability as well as emerging resistance with increased MICs, CI regimens of β-lactam antibiotics have been proposed to maximize time-dependent bacterial killing and, ultimately, to optimize treatment outcomes [6,7,8]. Despite the importance of clinical and pharmacodynamic evidence utilizing various administration methods based on real-world clinical pharmacokinetic profiles, there has been a relative paucity of data comparing the pharmacodynamic activities and clinical outcomes of C/T and CZA, the two preferred β-lactam antibiotic options available in Korea for the treatment of infections caused by CRPA, using CI versus intermittent-infusion (II) regimens.

Optimizing the antibiotic regimens for infections caused by CRPA is further complicated by the differences in the prevalent mechanisms of resistance depending on geographical regions [3,9]. The Antibacterial Resistance Leadership Group and Multi-Drug Resistant Organism Network Investigators reported no carbapenemase genes in 98% of CRPA in the United States, whereas the prevalence was only 68% and 43% in China and Singapore, respectively [9]. Such variability in resistance mechanisms may lead to differing antibiotic susceptibility profiles. According to a previous study, non-carbapenemase-producing CRPA isolates are susceptible to both C/T and CZA [10]. However, CRPA isolates producing carbapenemases show differential susceptibility [10]; while C/T was not active against Klebsiella pneumoniae carbapenemases (KPC)-producers, CZA demonstrated good activity against CRPA isolates harboring the majority of KPCs. Neither C/T nor CZA showed adequate in vitro activity against CRPA isolates producing New Delhi Metallo-β-lactamases (NDMs). Limited, variable information is available regarding the susceptibilities of carbapenemases more frequently detected outside Western countries, including Guiana Extended-Spectrum (GES) beta-lactamases, to C/T and CZA, leading to a lack of robust susceptibility data on prescribing information approved by regulatory agencies [11,12]. Consequently, these differences in antimicrobial susceptibility associated with resistance mechanisms make it extremely challenging to extrapolate preferred treatment options for various CRPA isolates with different genotypes. This emphasizes the need to consider both specific antibiotic-resistance genotypes and the prevalent mechanisms in a specific geographical area when selecting antibiotics, particularly for empiric therapy, for individual patients [9,13].

Although conventional in vitro time-kill studies have been frequently performed to assess antimicrobial susceptibility, they rely heavily on non-physiologic static systems where antimicrobial concentrations remain constant over time. While antibiotic concentrations may be physiologically mimicked by the supplementation or removal of medium, the bacterial population is also continually diluted, leading to inaccurate relationships between antibiotic concentrations and bacterial killing activity [14]. To address these challenges, the hollow fiber infection model (HFIM) has been increasingly utilized to investigate the in vitro pharmacodynamics of antibiotic agents in relation to their pharmacokinetics; this model simulates physiological pharmacokinetic profiles with serial assessments of drug exposure and relates antibiotic exposure to bacterial growth using different administration methods, such as varying doses and infusion times [15]. Therefore, the objectives of this study were to characterize the comparative pharmacodynamic profiles of C/T and CZA against a clinical CRPA isolate obtained from a patient encountered in clinical practice in Korea using the HFIM and to determine the pattern of resistance emergence in the tested CRPA isolate against C/T and CZA. Our findings may guide therapeutic decisions by further supporting the importance of genotype-specific treatment strategies for MDR organisms.

## 2. Materials and Methods

### 2.1. Antimicrobial Agents

Commercially available vials of ceftolozane/tazobactam (C/T; Zerbaxa^®^; Merck & Co., Inc., Rahway, NJ, USA; lot No. X015905; expiration date November 2025) and ceftazidime/avibactam (CZA; Avycaz^®^; Pfizer Inc., New York, NY, USA; lot No. 24K00058; expiration date July 2025) were used for both the HFIM and antimicrobial susceptibility testing. C/T and CZA were constituted in fixed ratios of 2:1 and 4:1, respectively. Comparators in the antimicrobial susceptibility testing, including imipenem and meropenem, were obtained as analytical-grade powders from Sigma-Aldrich (St. Louis, MO, USA). Their potency was adjusted according to certificates of analysis of the manufacturer.

Injection solutions of C/T and CAZ were prepared by reconstituting vials with sterile phosphate-buffered saline (PBS). Reconstituted solutions were stored at 2–8 °C in accordance with the manufacturer’s recommendation. No other agents were administered concomitantly with C/T or CZA.

### 2.2. Bacterial Strains and Culture Conditions

This study utilized two *Pseudomonas aeruginosa* strains: the reference strain ATCC 27853 (American Type Culture Collection, Manassas, VA, USA) as an antibiotic-susceptible control and a clinical isolate obtained from a patient during routine clinical practice at Kyung Hee University Medical Center (Seoul, Republic of Korea) as a drug-resistant test organism. The single clinical isolate was identified as a carbapenem-resistant *P. aeruginosa* (CRPA) harboring a GES-type carbapenemase, confirmed via peptide nucleic acid-mediated multiplex real-time PCR (PANA Real Typer CRE kit; PANAGENE Inc., Daejeon, Republic of Korea) targeting *β*-lactam-resistance genes. Peptide nucleic acid-mediated multiplex real-time PCR was performed using primers and probes designed to screen for five major carbapenemase gene families (IMP, VIM, NDM, KPC, and GES) and an internal control provided by PANAGENE, Inc.; the sequences have been reported previously [16]. The sensitivity, specificity, and detection limit of the multiplex real-time PCR assay (PANA Real Typer CRE kit; PANAGENE Inc., Daejeon, Republic of Korea) were 100%, 99.8%, and 10 copies per 20 μL of reaction volume, respectively [17,18]. Multiplex real-time PCR was performed in a 96-well plate using the CFX-96 Real-Time PCR detection system (Bio-Rad Laboratories Inc., Hercules, CA, USA), according to the manufacturer’s instructions. Reaction mixtures comprised 19 μL of primer/probe and multiplex real-time PCR master mixture, 5 μL of genomic DNA, and 1 μL of Taq DNA polymerase. After each run, the threshold cycle (Ct) was measured based on the signal strength at which the fluorescence exceeded the threshold. Samples with Ct values < 35 were considered positive. RNase-free water was included as a negative control in each run. The reference strain ATCC 27853 was also subjected to this analysis to screen for the presence of resistance genes.

Bacterial stocks were preserved at −80 °C using Cryobeads (BioMérieux, Marcy-l’Étoile, France) according to the manufacturer’s instructions. For experimental preparation, beads were streaked onto Mueller–Hinton agar (MHA; Sigma-Aldrich, St. Louis, MO, USA) and incubated overnight at 37 °C to obtain isolated colonies. A single isolated colony was subsequently inoculated into Mueller–Hinton broth (MHB; Sigma-Aldrich, St. Louis, MO, USA) and cultured at 37 °C with shaking at 180 rpm to log-phase growth. The bacterial suspension was adjusted to a target density of approximately 10^7^–10^8^ CFU/mL based on optical density at 600 nm (OD_600_). The final inoculum size was verified by quantitative culture on a drug-free MHA plate.

### 2.3. Antibiotic Susceptibility Testing

MIC values were determined by broth microdilution in MHB following the guidelines of the Clinical and Laboratory Standards Institute (CLSI M07) [19]. To ensure susceptibility data were biologically representative for pharmacodynamic simulations, the inoculum used for MIC determination was prepared by diluting the bacterial suspension reconstituted for the HFIM to a final density of approximately 5 × 10^5^ CFU/mL. The reference strain *P. aeruginosa* ATCC 27853 was included as a quality control in all susceptibility testing runs. The modal MIC values were used. For the combination agents, C/T and CZA, susceptibility testing was performed with tazobactam and avibactam concentrations fixed at 4 mg/L. Carbapenem-resistance profiles were confirmed by determining the MICs of imipenem and meropenem via broth microdilution. Susceptibility profiles for other comparator agents (i.e., cephalosporins, monobactam, and fluoroquinolones) were assessed using an automated system (VITEK 2, bioMérieux, Marcy-l’Étoile, France). MIC results were interpreted according to the current CLSI breakpoints (M100) [20].

### 2.4. Hollow Fiber Infection Model Setup and Inoculation

The HFIM was utilized to simulate clinically relevant pharmacokinetic profiles of C/T and CZA and to evaluate changes in bacterial growth over time in triplicate independently with continuous aseptic integrity monitoring of the HFIM system. Polysulfone cartridges (C2011; FiberCell Systems Inc., Frederick, MD, USA) were inoculated by introducing 20 mL of a log-phase bacterial suspension (10^7^–10^8^ CFU/mL) into the extracapillary space (ECS). While we did not experimentally evaluate drug adsorption to the hollow fiber membranes in this study, polysulfone was selected as the membrane material because the agents tested in this study have been reported to exhibit negligible non-specific binding or adsorption to polysulfone fibers [21]. The cartridge was connected to a central reservoir and maintained at 37 °C in an incubator. Fresh, drug-free MHB was continuously supplied to the central reservoir to simulate drug elimination and provide nutrients, while an efflux pump removed waste and excess medium to maintain a constant volume. The media, including antimicrobial agents in the central reservoir contents, were rapidly equilibrated with the ECS via a Duet pump (FiberCell Systems Inc., Frederick, MD, USA).

#### 2.4.1. Pharmacokinetic Simulation and Experimental Design of HFIM

Pharmacokinetic parameters used to simulate plasma drug concentration–time profiles in the HFIM were derived from the prescribing information of each product in patients with indicated infectious diseases [22,23]. Unbound plasma concentrations of ceftolozane and ceftazidime were estimated by accounting for the protein-binding fraction of each drug (21% for ceftolozane; 10% for ceftazidime) as stated in the prescribing information approved by the FDA [22,23]. The HFIM simulated the intermittent infusion (II) regimens approved by global regulatory agencies such as the Food and Drug Administration (FDA), the European Medicines Agency (EMA), or the Ministry of Food and Drug Safety (MFDS) as follows: C/T 1.5 g (1 g/0.5 g) infused over 1 h every 8 h and CZA 2.5 g (2 g/0.5 g) infused over 2 h every 8 h. The target maximum unbound concentrations (*f*C_max_) for II regimens were 58.8 mg/L for ceftolozane and 81.4 mg/L for ceftazidime [22,23]. All drugs were infused every 8 h through programmable syringe pumps (New Era Pump System, Inc., Farmingdale, NY, USA) into the central reservoir to achieve plasma drug exposures in humans based on the concentrations of *β*-lactam components (i.e., ceftolozane and ceftazidime). Additionally, the same total daily dosages of each drug were simulated in the HFIM as CI regimens administered by the first (i.e., loading) dose infused over 30 min followed by constant infusion to achieve the steady-state average concentrations (*f*C_ss,avg_) of CTZ and CAZ calculated using the pharmacokinetic parameters and the approved II dosing regimens. The CI regimens were administered into the central reservoir via programmable syringe pumps (New Era Pump System, Inc., USA). The flow rate of diluent media was fixed throughout the experiments to reproduce the human half-life of 3.0 h for ceftolozane. Precise simulation of a shorter half-life for tazobactam (i.e., 1 h) was not considered necessary due to its limited activity against *P. aeruginosa* [24,25]. Likewise, the flow rate of diluent media was maintained at the human half-life of 2.7 h for both ceftazidime and avibactam as suggested in the prescribing information of CZA [23]. The target pharmacokinetic parameters used to simulate human exposure profiles in the HFIM are summarized in Table 1. A total of six experimental arms were tested as follows: (1) the standard strain ATCC 27853 treated with C/T infused over 1 h every 8 h; (2) the standard strain ATCC 27853 treated with CZA infused over 2 h every 8 h; (3) the clinical CRPA isolate treated with C/T infused over 1 h every 8 h; (4) the clinical CRPA isolate treated with C/T continuously infused following 30 min infusion of a loading dose; (5) the clinical CRPA isolate treated with CZA infused over 2 h every 8 h; (6) the clinical CRPA isolate treated with CZA continuously infused following 30 min infusion of a loading dose.

#### 2.4.2. Pharmacodynamic Analysis and Bacterial Quantification

Samples were serially collected from the ECS at 0 (pre-dose), 8, 24, 48, 72, 96, 120, 144, and 168 h to determine the bactericidal activity of the tested drugs (i.e., C/T and CZA) over the 7-day treatment period [3,26]. For experimental arms exhibiting undetectable bacterial growth by 168 h (i.e., 7 days) at the time of discontinuing antibiotic infusion, additional sampling was performed at 192 and 216 h to monitor for potential regrowth or confirm eradication of the bacterial strain. To minimize drug carryover, all collected samples were washed twice by centrifugation at 157,000× *g* for 3 min. The pellets were resuspended, serially diluted, and plated on MHA to quantify bacterial density (log_10_ CFU/mL). The lower limit of detection was 20 CFU/mL (1.3 log_10_ CFU/mL).

### 2.5. Population Analysis Profiles Assay

To characterize the pattern of resistance emergence in the tested bacterial population, quantitative cultures were performed on drug-supplemented agar plates. Samples collected from the HFIM at 48 h, 96 h, and 168 h were serially diluted and plated onto MHA supplemented with C/T or CZA at concentrations corresponding to three times the baseline MIC (3 × MIC), 6 × MIC, and 9 × MIC. The baseline MIC values used in this experiment were determined in the antimicrobial susceptibility testing (Section 2.3). This stratification was used to screen for the selection of subpopulations with low, moderate, and high levels of resistance, respectively [27,28]. Unlike standard susceptibility testing where inhibitor concentrations are fixed (e.g., 4 mg/L), the ratio of the *β*-lactam to the *β*-lactamase inhibitor was maintained as constant (2:1 for C/T and 4:1 for CZA) across all tested concentrations to reflect the commercial formulations used in the HFIM. Inoculated plates were incubated for 24 h at 37 °C, and colonies were counted to quantify the density of the resistant subpopulation (log_10_ CFU/mL). All experiments were performed in triplicate. To confirm the resistance phenotypes of the surviving subpopulations, representative single bacterial colonies were isolated from the population analysis profile (PAP) agar plates. The MICs of these recovered isolates were subsequently re-evaluated using the standard antimicrobial susceptibility testing methods described previously to verify any stable increases in MIC values.

### 2.6. Statistical Analysis

All bacterial densities were log_10_-transformed prior to statistical analysis. Changes in bacterial densities from the initial inoculum (0 h) were evaluated using one-way analysis of variance (ANOVA) followed by Dunnett’s multiple comparisons test. Comparisons of resistance emergence between intermittent and continuous infusion regimens across specific time points were performed using two-way ANOVA with Bonferroni’s correction. Statistical analyses were performed using GraphPad Prism software (version 9.0; GraphPad Software, San Diego, CA, USA). A *p*-value of <0.05 was considered statistically significant.

## 3. Results

### 3.1. Bacterial Genotyping and Antibiotic Susceptibility

The results of genotyping analyses and antibiotic susceptibility testing for the standard strain (ATCC 27853) and the clinical CRPA isolate are summarized in Table 2. Genotyping analyses confirmed the clinical isolate harbored the *bla_GES_* carbapenemase gene, whereas the standard strain was negative for carbapenemase genes.

Phenotypically, the clinical isolate was characterized as MDR and carbapenem-resistant. Susceptibility testing results based on both broth microdilution and automated systems revealed high-level resistance across major antimicrobial classes. Specifically, the isolate exhibited high-level resistance to carbapenems with MICs of 64 mg/L for imipenem and 256 mg/L for meropenem. In addition, it was not susceptible to most of the comparators including cephalosporins, monobactam, and fluoroquinolones. For the testing agents including C/T and CZA, the clinical isolate exhibited intermediate susceptibility to C/T, but remained fully susceptible to CZA. In contrast, the standard strain was susceptible to both agents.

### 3.2. HFIM Study Using the Standard ATCC 27853 Strain

**Figure 1:**
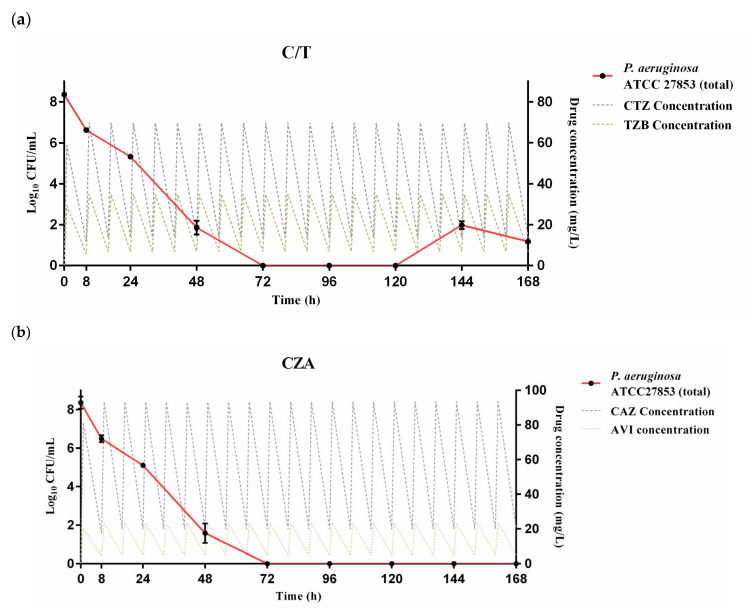
Activities of approved intermittent-infusion regimens of (**a**) ceftolozane/tazobactam (C/T) and (**b**) ceftazidime/avibactam (CZA) against the standard control strain *Pseudomonas aeruginosa* ATCC 27853 in the HFIM. The total bacterial densities (left *Y*-axis, solid red line with black filled circles, log_10_ CFU/mL) and simulated free drug concentration (right *Y*-axis, dashed lines, mg/L) are shown. A significant reduction in bacterial densities from the initial inoculum was observed for both C/T and CZA at all time points starting from 8 h (*p* < 0.01). Error bars indicate the standard deviation (SD) estimated by triplicate experiments. The limit of detection (LOD) was 1.3 log_10_ CFU/mL. Abbreviations: ATCC, American Type Culture Collection; CTZ, ceftolozane; TZB, tazobactam; CAZ, ceftazidime; AVI, avibactam; CFU, colony-forming unit.

The pharmacodynamic profiles of C/T and CZA against the standard quality control strain (ATCC 27853) are shown in Figure 1. Both agents, administered via intermittent infusions (i.e., 1 and 2 h infusion for C/T and CZA, respectively), demonstrated rapid and potent antibacterial effects from early on during the treatment period. With the initial inoculum of 8.4 log_10_ CFU/mL, bacterial densities at the 8 h time point were reduced by 1.77 log_10_ CFU/mL in the C/T treatment group and 1.93 log_10_ CFU/mL in the CZA group. The antibacterial activity of both agents was potentiated over time. Bactericidal activity (defined as ≥3 log_10_ CFU/mL reduction from baseline) was achieved by 48 h, with reductions in bacterial burden by 6.46 log_10_ CFU/mL for C/T- and 6.71 log_10_ CFU/mL for CZA-treated strains. Bacterial growth inhibition persisted until the end of the 7-day experimental period (i.e., 168 h) for both drugs. In the CZA arm, bacterial counts dropped below the limit of detection by 72 h and remained undetectable until the end of the experiment (Figure 1b). In the C/T-challenged strain, a transient rebound in bacterial density to 2.01 log_10_ CFU/mL was observed at 144 h; however, this was suppressed by 168 h (Figure 1a). Samples collected from the C/T-treated arm at 168 h yielded no viable colonies on drug-free MHB, confirming the eradication of the standard strain without the emergence of resistant subpopulations.

### 3.3. HFIM Studies Using the Clinical CRPA Isolate Treated with C/T and CZA Administered by Intermittent Infusions

**Figure 2:**
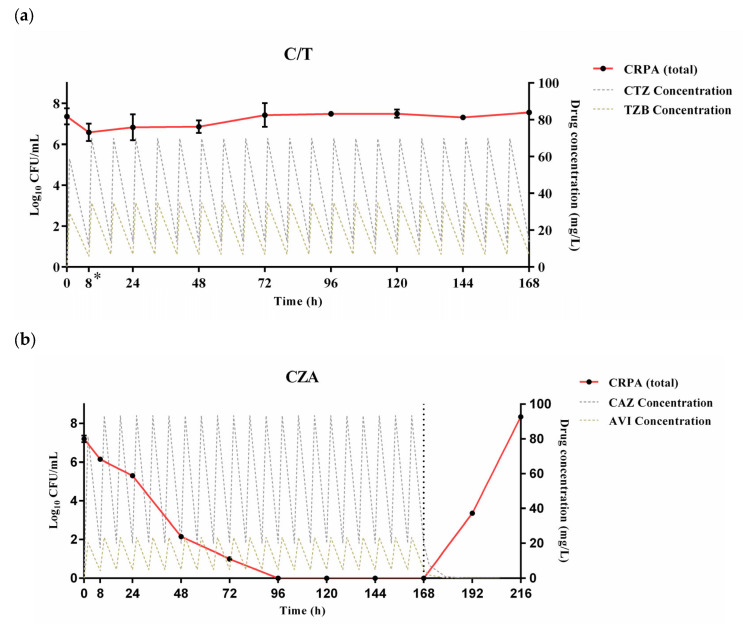
Activities of approved intermittent-infusion regimens of (**a**) ceftolozane/tazobactam (C/T) and (**b**) ceftazidime/avibactam (CZA) against the clinical isolate of carbapenem-resistant *Pseudomonas aeruginosa* (CRPA). The total bacterial densities (left *Y*-axis, solid red line with black filled circles, log_10_ CFU/mL) and simulated free drug concentration (right *Y*-axis, dashed lines, mg/L) are shown. The GES-type carbapenemase was detected in the tested CRPA isolate. The vertical dotted line denotes the cessation of CZA infusion at 168 h. Error bars indicate the standard deviation (SD) estimated by triplicate experiments. A single asterisk (*) in (**a**) indicates a significant bacterial reduction from the initial inoculum at 8 h only (*p* < 0.05). A significant reduction in bacterial densities from the initial inoculum was observed with CZA at all time points starting from 8 h until 168 h (*p* < 0.05) (**b**). Abbreviations: CTZ, ceftolozane; TZB, tazobactam; CAZ, ceftazidime; AVI, avibactam; CFU, colony-forming unit.

Figure 2 represents the time courses of the bacterial burden for the clinical CRPA isolate producing the GES-type carbapenemase treated with C/T and CZA administered via intermittent infusions. When the clinical CRPA isolate was challenged with simulated human exposures of C/T (1 h infusion) at an initial inoculum of 7.37 log_10_ CFU/mL, no significant reduction in bacterial density was observed throughout the 7-day experimental period (Figure 2a). The bacterial burden fluctuated within ±1 log_10_ CFU/mL of the initial inoculum over 168 h (*p* > 0.05 for all observed time points except 8 h), demonstrating the lack of bactericidal activity of C/T against the GES-producing clinical CRPA isolate.

In contrast, the II regimen of CZA (i.e., 2 h infusion) demonstrated rapid antibacterial activity within 8 h, which persisted throughout the antibiotic treatment period up to 168 h (Figure 2b). From the initial inoculum of 7.22 log_10_ CFU/mL, bacterial density decreased by 1.05 log_10_ CFU/mL at 8 h and further by 1.92 log_10_ CFU/mL at 24 h. This antibacterial activity was enhanced over time, achieving bactericidal thresholds (≥3 log_10_ reduction) by 48 h with a reduction by 5.07 log_10_ CFU/mL. No viable bacterial colonies were detected from 72 h to 168 h. However, bacterial growth inhibition did not persist after cessation of antibiotic administration. Following discontinuation of CZA administration at 168 h, significant bacterial regrowth occurred; bacterial density rebounded exponentially to 8.35 log_10_ CFU/mL by 216 h (i.e., 48 h after withdrawal of antibiotic therapy), exceeding the initial inoculum size.

### 3.4. HFIM Studies Using the Clinical CRPA Isolate Treated with C/T and CZA Administered by Continuous Infusions

**Figure 3:**
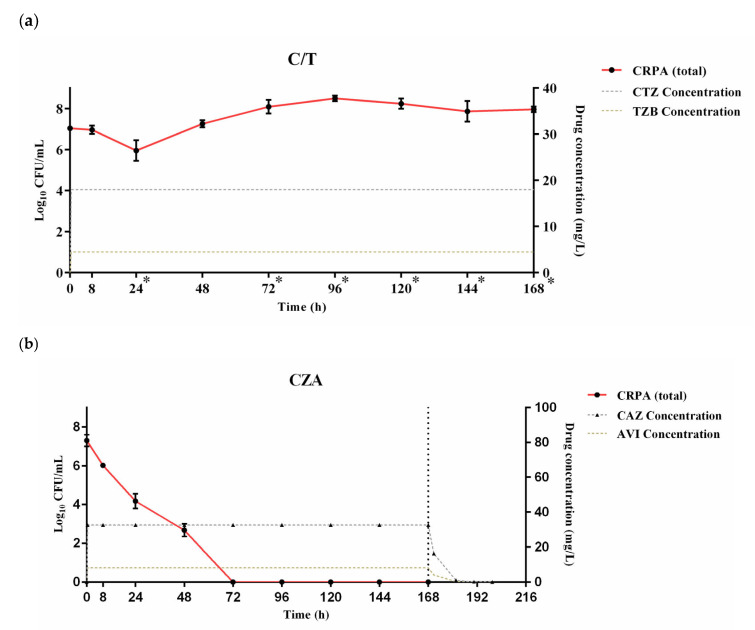
Activities of continuous-infusion regimens of (**a**) ceftolozane/tazobactam (C/T) and (**b**) ceftazidime/avibactam (CZA) against the clinical isolate of carbapenem-resistant *Pseudomonas aeruginosa* (CRPA). The total bacterial densities (left *Y*-axis, red line with black filled circles, log_10_ CFU/mL) and simulated free drug concentration (right *Y*-axis, dashed lines, mg/L) are shown. The GES-type carbapenemase was detected in the tested CRPA isolate. For both C/T and CZA, loading dose was administered by a single infusion over 30 min concurrently with the initiation of continuous infusion. Treatment was discontinued at 168 h (i.e., 7 days) with additional bioburden data collected up to 216 h in the CZA-treated arm. Error bars indicate the standard deviation (SD) estimated by triplicate experiments. A single asterisk (*) in (**a**) indicates a significant change in bacterial densities from the initial inoculum at the indicated time point (*p* < 0.05). A significant reduction in bacterial densities from the initial inoculum was observed with CZA at all time points starting from 8 h until 168 h (*p* < 0.05) (**b**). Abbreviations: C/T, ceftolozane/tazobactam; CZA, ceftazidime/avibactam; CTZ, ceftolozane; TZB, tazobactam; CAZ, ceftazidime; AVI, avibactam; CFU, colony-forming unit; CRPA, carbapenem-resistant *Pseudomonas aeruginosa*.

Figure 3 shows bacterial populations of the clinical CRPA isolate producing the GES-type carbapenemase over time against C/T and CZA administered via continuous infusions for 7 days (i.e., 168 h). When the clinical CRPA isolate was challenged with the CI regimen of C/T to maintain the target *f*C_ss,avg_ following the loading dose infused over 30 min, a significant reduction in bacterial density was observed from the initial inoculum of 7.04 log_10_ CFU/mL, with a reduction of 0.9 log_10_ CFU/mL (*p* < 0.05) at 24 h. Subsequently, from 72 h onwards, the bacterial burden was increased significantly until the end of the experiment (i.e., 168 h) (Figure 3a). The bacterial load was maintained at ±1.5 log_10_ CFU/mL from the initial inoculum size over 168 h, demonstrating the lack of bactericidal activity of C/T against the GES-type CRPA.

For the CI regimen of CZA, antibacterial effect was observed within 8 h of initiating treatment and subsequently maintained throughout the antibiotic treatment period up to 168 h (Figure 3b). From the initial inoculum of 7.22 log_10_ CFU/mL, bioburdens were reduced by 1.37 log_10_ CFU/mL at 8 h and further decreased by 2.97 log_10_ CFU/mL at 24 h. Afterward, bactericidal activity was achieved at 48 h with a decrease in bacterial load by 4.48 log_10_ CFU/mL. No bacterial colony was visibly detected from 72 h to 168 h. Upon discontinuation of 7-day treatment with CZA at 168 h, no bacterial regrowth was observed by 216 h (i.e., 48 h after withdrawal of antibiotic therapy), indicative of the sustained dormant state of the pathogen even after cessation of the CI regimen of CZA.

Overall, neither the II regimen nor the CI regimen of C/T showed bactericidal activity against the GES-type CRPA isolate throughout the 7-day treatment period. Both the II and the CI regimens of CZA achieved bactericidal effects during the 7 days of therapy; bacterial regrowth inhibition persisted post-treatment only with the CI regimen.

### 3.5. Population Analysis Profiles

**Figure 4:**
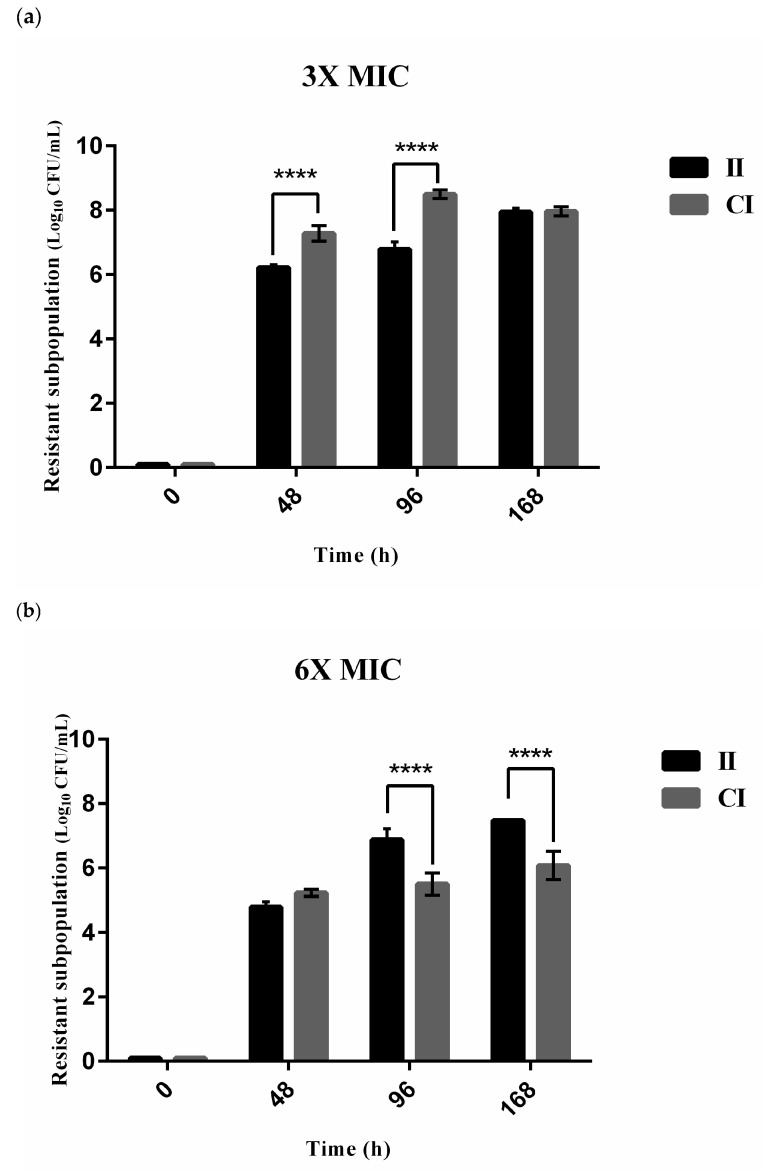

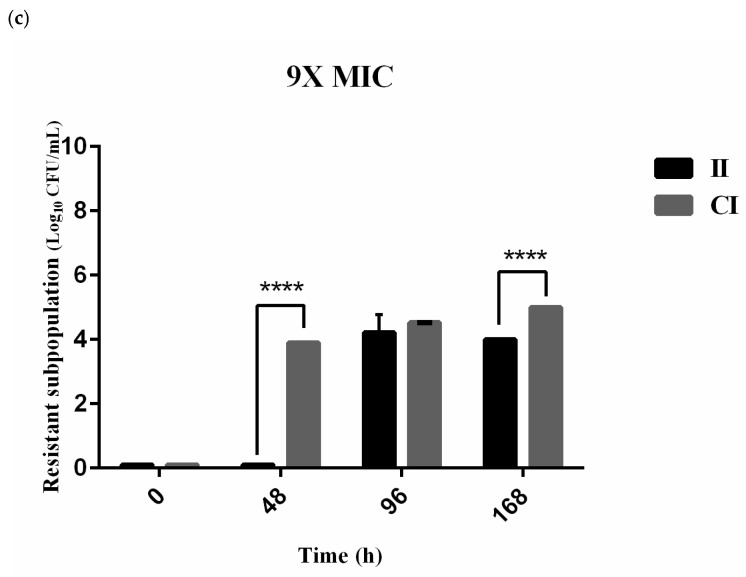
Emergence of resistant subpopulations for the GES-type clinical CRPA isolate treated with ceftolozane/tazobactam (C/T) administered by intermittent and continuous infusion at 48 h, 96 h, and 168 h of therapy on the (**a**) 3 × MIC (24 mg/L), (**b**) 6 × MIC (48 mg/L), and (**c**) 9 × MIC (72 mg/L) C/T agar plates. Black and grey bars represent the mean densities of the resistant subpopulation (log_10_ CFU/mL) for the II and CI regimens, respectively. Error bars indicate the standard deviation (SD) from triplicate experiments. Four asterisks (****) indicate a statistically significant difference (*p* < 0.0001) between the II and CI regimens at the respective time point. Abbreviations: CFU, colony-forming unit; MIC, minimum inhibitory concentration; II, intermittent infusion; CI, continuous infusion.

In the C/T treatment arm (Figure 4), subpopulations with elevated C/T MICs, indicative of resistant subpopulations, were detected in both the II- and CI-regimen groups. On the 3 × MIC C/T plates (Figure 4a), C/T-resistant colonies arose in greater densities in the CI-challenged group compared to those with the II group at 48 h and 96 h of C/T treatment (*p* < 0.0001). However, the subpopulation culture reached similar densities at 168 h (7.95 and 7.98 log_10_ CFU/mL in the II and CI group, respectively; *p* > 0.05). On the 6 × MIC plates (Figure 4b), comparable densities of resistant subpopulations from inoculum were detected between the II and CI groups at 48 h. However, significant growth of resistant colonies was detected only in the II group at 96 h and 168 h (*p* < 0.0001); the resistant subpopulation was maintained at a comparable burden in the CI group. On the 9 × MIC plates (Figure 4c), the resistant subpopulation of the clinical isolate challenged with the CI regimen was detected in similar densities at all measured time points (i.e., 48 h, 96 h, and 168 h). Treatment-emergent resistance appeared more slowly in the II group at significantly lower densities compared to the CI group (*p* < 0.0001); a resistant subpopulation was not detected at 48 h, and then reached 4.0 log_10_ CFU/mL at 168 h.

In contrast, for the clinical isolates treated with CZA, no subpopulations with elevated CZA MICs arose in either the II- or CI-regimen group. Throughout the 168 h experiments, treatment-emergent resistance was not detected on any of the 3×, 6×, and 9 × MIC agar plates.

## 4. Discussion

To our knowledge, this is one of the first few studies comparatively evaluating the pharmacodynamics of C/T and CZA against clinically encountered CRPA with *bla_GES_* in the HFIM using different infusion methods, i.e., the II and CI regimen. Although previous studies investigated their spectrum of activities against various pathogens, published data are relatively scarce regarding the antibacterial activities of C/T and CZA against the MDR organisms mostly endemic in non-US countries, including those producing GES-type carbapenemase [9,29]. Our tested CRPA with GES-type carbapenemase showed MICs of 8/4 mg/L for both C/T and CZA (Table 2), interpreted as intermediate (I) and susceptible (S), respectively [19]. Considering prolonged or continuous infusion might improve the bactericidal activity of beta-lactam antibiotics through maximizing *f*T > MIC against less susceptible organisms [30,31], we evaluated the in vitro efficacy of C/T and CZA against the tested clinical isolate using II and CI regimens. Consistent with previous studies reporting the tolerability and possibly improved efficacy of CZA administered by the CI regimen for the treatment of infections caused by various MDR pathogens with other mechanisms of resistance than GES-type carbapenemases [32,33], our present study suggested the potential therapeutic advantage of the CI CZA regimen over tested regimens for treatment of GES-type CRPA infections due to complete eradication confirmed by no bacterial regrowth following antibiotic discontinuation as well as excellent bactericidal activity during 7 days of therapy.

Over the last decade, a few antimicrobial agents have been newly approved for infections caused by MDR Gram-negative organisms with ESBLs or carbapenemases [3]. The most recent clinical practice guideline published by the IDSA regarding the treatment of MDR Gram-negative infections recommended imipenem/cilastatin/relebactam, C/T, and CZA as preferred options for the treatment of systemic infections outside the urinary tract [3]; among them, imipenem/cilastatin/relebactam is not yet approved in Korea. However, as acknowledged in the current IDSA guideline [3], clinical trials are lacking to compare novel antibiotic agents to each other such as C/T vs. CZA. Considering the potential differences in the antibiotic susceptibilities of CRPA due to regional variability in mechanisms of resistance, we investigated the comparative pharmacodynamic activities of C/T and CZA against the CRPA isolate clinically encountered in Korea. The resistance gene revealed in our tested clinical isolate was GES-type beta-lactamase (*bla*_GES_), which is not clearly stated in the approved label of either antibiotic agent to render C/T or CZA effective or ineffective. Therefore, our current study may contribute to the current body of literature through comparing the pharmacodynamic activities of C/T and CZA against CRPA with *bla*_GES_ frequently encountered in non-US endemic areas such as Korea [9,29], suggesting CZA as a potentially preferable treatment option to C/T for systemic infections caused by CRPA with *bla*_GES_ or in areas where the emergence of *bla*_GES_-harboring pathogens is nonnegligible.

Our present study confirmed previous study findings suggesting CZA as an efficacious treatment option against the clinical CRPA isolate with *bla*_GES_ [34]. This efficacy of CZA is primarily driven by the potent, non-β-lactam inhibitory activity of avibactam, which provides robust and reversible inhibition of GES-type carbapenemases, thereby effectively protecting ceftazidime from enzymatic hydrolysis. With 7 days of the II CZA regimen using target concentration profiles equivalent to the approved dosing (i.e., 2 g/0.5 g infused over 2 h every 8 h), bactericidal activity was attained at 48 h without detectable bacterial colonies from 96 h (Figure 2). However, exponential bacterial regrowth occurred after discontinuation of II therapy, potentially indicating incomplete eradication and subsequent proliferation after 7 days of therapy. This post-treatment regrowth was not observed with 7 days of the CI regimen, suggesting complete eradication of the tested GES-type CRPA (Figure 3). The post-exposure bacterial regrowth might raise concern regarding in vivo evolution of resistance to CZA through genetic mutations as suggested by previous publications [35,36]. However, our PAP results demonstrated the absence of resistant subpopulations selected or proliferated after 7 days of II or CI therapy with CZA. In the absence of resistant subpopulations harboring newly emerging genetic mutations, the rapid bacterial regrowth immediately after 7 days of CZA therapy administered by the II regimen might be accounted for by incomplete eradication of GES-type CRPA as well as survival of persister cell populations or transient phenotypic tolerance. Future studies might be warranted to explore the exact mechanisms of rapid bacterial regrowth after discontinuation of intermittent-infusion CZA therapy and to investigate if extending the duration of therapy (e.g., 14 days) beyond 7 days minimizes the risk of post-antibiotic bacterial regrowth. Overall, the CI regimen of CZA might be considered for complete eradication of CRPA with *bla*_GES_ to maximize treatment outcomes for infections caused by MDR *P. aeruginosa*, particularly when the infection risk for GES-type CRPA is reasonably high.

In contrast to CZA, C/T was not effective in inhibiting the growth of CRPA with *bla*_GES_ (Figure 2 and Figure 3). The CI regimen of C/T failed to enhance antibacterial activity against our tested GES-type CRPA isolate (Figure 3). Our current study findings were consistent with previous study results suggesting the intrinsic resistance of GES-type carbapenemases, particularly GES-5 or 6, to C/T in *P. aeruginosa* and Enterobacterales through hydrolyzing cephalosporins and bypassing the inhibitory activity of tazobactam on carbapenemase [10,37]. Moreover, our PAP results revealed exposure of the GES-type CRPA to C/T resulted in promoting the selection and proliferation of resistant subpopulations (Figure 4). When exploring the change in resistant subpopulation densities at 48 h, 96 h, and 120 h, the lowest growth rate was observed for the subpopulation with a high level of resistance (i.e., 9 × MIC), followed by that with a moderate (i.e., 6 × MIC) and then with a low level of resistance (i.e., 3 × MIC). The slower growth rate might be a biological cost for the enhanced survival benefit granted to highly resistant organisms [38]. Compared to the II regimen, the CI regimen was associated with a relatively higher prevalence of resistant subpopulations (Figure 4), potentially due to the maintenance of constant antibiotic exposures providing an environment to favor the growth of resistant subpopulations through effectively eliminating susceptible strains from survival competition. This possibly detrimental effect of the CI regimen of C/T on resistance emergence should be further investigated in future large-scale studies using various antibiotics tested against multiple bacterial strains.

There are limitations to be addressed in this study. First, only a single GES-type CRPA isolate was utilized to comparatively evaluate the antibacterial activity of CZA and C/T administered by different infusion methods, potentially limiting the generalizability and robustness of our study findings. While CRPA with *bla*_GES_ has recently been considered as a growing concern implicated in infections caused by MDR organisms in some geographical areas such as Japan and Korea [39], other types of CRPA isolates including those carrying *bla_NDM_*, *bla_KPC_*, and efflux pump genes (e.g., *oprD*) are also commonly encountered in clinical practice globally. Although the multiplex real-time PCR assay in our study tested the presence of five major carbapenemase gene families, i.e., IMP, VIM, NDM, and KPC, as well as GES, the co-existence of other carbapenem-resistance genes could not be confirmed without performing whole-genome sequencing. Moreover, a specific subtype of *bla*_GES_ was not characterized in our clinical CRPA isolate. Nonetheless, the antimicrobial susceptibility test results might inform the possible subtype of GES carbapenemase in our tested isolate. Still, due to the unknown genotype of *bla*_GES_ in our tested CRPA isolate, caution should be exercised when interpreting our present study findings. Also, the relatively sparse sampling schedule employed in our present study simply reported the attainment of a bactericidal threshold at pre-specified discrete time points rather than continuously estimating the model-informed time to reach bactericidal effects. Comparative interpretation of pharmacodynamic activities for CZA and C/T might be enhanced with the model-informed time and exposures to achieve bactericidal activity using quantitative pharmacology modeling. Furthermore, only the representative pharmacokinetic profiles of CZA and C/T in typical patients with indicated infections were simulated in our HFIM without considering interpatient pharmacokinetic variability. Additionally, our HFIM simulated free drug concentrations in human plasma. Because actual drug concentrations at specific infection sites (e.g., epithelial lining fluid in the lungs) can be lower due to variable tissue penetration, the simulated plasma exposures might overestimate target-site pharmacokinetics, which could influence actual clinical outcomes. Notably, the simulated antibiotic concentrations were not validated with direct measurement of concentrations in the system over time; rather, they relied solely on the rigorous, highly controlled system of HFIM to reproduce the desired drug exposures. Future validation of the PK model would further enhance the reliability and robustness of our study findings. In addition, mutation frequency assays were not performed in the present study, leading to a lack of knowledge regarding spontaneous resistance emergence rates at baseline. Consequently, our PAPs showed the overall impact of C/T treatment administered by II or CI regimens on the emergence of resistant subpopulations over time without adjusting for the spontaneous resistance emergence rates. Thus, caution should be exercised when interpreting our study findings as the sole effects of C/T exposures on the emergence of resistant CRPA subpopulations. Lastly, the HFIM does not replicate the contribution of the host immune system to treatment outcomes of bacterial infections. The interaction between antibiotic therapy and the host immune system is a critical determinant of the ultimate treatment outcome [40]; consequently, the lack of immune system components in the HFIM might result in a conservative prediction of the clinical response to antibiotic therapy. Furthermore, our present HFIM study focused exclusively on monotherapy with C/T and CZA. Although the most recent international treatment guidelines suggested C/T or CZA monotherapy as a first-line treatment option for CRPA infections [3], combination therapy is frequently utilized in clinical practice for severe MDR infections to maximize efficacy and prevent resistance. Future dynamic model studies evaluating combination regimens are warranted to more diverse and complex clinical decisions. Further clinical studies are warranted to optimize antibiotic therapy for infections caused by CRPA and other MDR bacterial organisms characterized by whole-genome sequencing through suggesting model-informed exposures and time to achieve bactericidal effects with dense sampling schedules, especially over the initial phase of antibiotic therapy, and ultimately, validating our current study findings in various real-world patient populations.

## 5. Conclusions

In conclusion, CZA showed bactericidal activity in vitro against the tested CRPA isolate with *bla*_GES_ in the rigorous, highly controlled HFIM. However, the II regimen of CZA resulted in rapid regrowth of GES-type CRPA after discontinuation of 7-day therapy. When administered by the CI regimen, 7-day treatment with CZA maintained sustained suppression of bacterial growth without detectable regrowth after treatment cessation. No significant antibacterial activity was observed with C/T in vitro against the tested GES-type CRPA. Resistance to C/T rapidly developed in GES-type CRPA after initiation of therapy with a relatively higher density of resistant subpopulations emerging when treated with the CI regimen compared to the II regimen. Based on our HFIM studies, CZA might be a valuable therapeutic option against infections caused by similar GES-producing CRPA, particularly when administered by continuous infusion to prevent the emergence of resistance as well as to treat the ongoing infection. Future studies, such as in vitro evaluations with a broader diverse collection of CRPA isolates using dense sampling schedules as well as clinical studies, are warranted to confirm the comparative effectiveness of the II and CI regimen of CZA in patients infected by CRPA isolates, including those with *bla*_GES_.

## Figures and Tables

**Table 1:** Pharmacokinetic parameters and target concentrations simulated in the hollow fiber infection model (HFIM) ^a,b^.

Test Product ^c^	Ingredient	Half-Life (*t*_1/2_, h) ^d^	Protein Binding (%)	Regimen ^e^	Dosing Schedule	*f*C_max,ss_ (mg/L)	*f*C_min,ss_ (mg/L)	*f*C_ss,avg_ (mg/L)
Ceftolozane/ tazobactam (1 g/0.5 g)	Ceftolozane	3.0	21	II	Q8h, 1 h infusion	69.80	13.85	-
				CI	30 min loading dose + CI	-	-	32.74
	Tazobactam		-	II	Q8h, 1 h infusion	34.90	6.93	-
				CI	30 min loading dose + CI	-	-	16.37
Ceftazidime/ avibactam (2 g/0.5 g)	Ceftazidime	2.7	10	II	Q8h, 2 h infusion	93.38	20.01	-
				CI	30 min loading dose + CI	-	-	17.97
	Avibactam		-	II	Q8h, 2 h infusion	23.34	20.01	-
				CI	30 min loading dose + CI	-	-	4.49

^a^ All concentration values represent free (unbound to plasma proteins) fractions of the drug. Free drug concentrations were calculated based on protein-binding rates of 21% for ceftolozane and 10% for ceftazidime. ^b^ II, intermittent infusion; CI, continuous infusion; Q8h, every 8 h; *f*C_max,ss_, free drug maximum concentration at steady state; *f*C_min,ss_, free drug minimum concentration at steady state; *f*C_ss,avg_, free drug steady-state average concentration for continuous infusion. ^c^ Pharmacokinetic profiles for the beta-lactamase inhibitor (i.e., tazobactam and avibactam) were simulated based on the fixed ratios of each product (i.e., 2:1 for ceftolozane/tazobactam and 4:1 for ceftazidime/avibactam). ^d^ In the HFIM system, tazobactam and avibactam concentrations were not separately simulated; their concentrations were co-simulated simply based on the half-life of the beta-lactam agent combined with them in the product because fixed-ratio commercially available products were used in this study (i.e., 3.0 h for ceftolozane and 2.7 for ceftazidime). ^e^ Continuous infusion regimens started with a loading dose administered over 30 min to rapidly achieve the target steady-state concentrations.

**Table 2:** Genotypic characteristics and antibiotic susceptibility of the tested strains ^a,c^.

Strain			Beta-Lactamase (*bla*) Genes
ATCC 27853			None detected
Clinical strain			*bla_GES_*
**Susceptibility Testing Result: Standard Strain (ATCC 27853)**			
**Antibiotic**			**MIC (mg/L) (Interpretation) ^b^**
Study Drugs	C/T		<1/4 (S)
	CZA		<1/4 (S)
**Susceptibility Testing Result: Clinical Strain (with *bla_GES_*)**			
**Antibiotic**			**MIC (mg/L) (interpretation) ^b^**
Study Drugs		C/T	8/4 (I)
		CZA	8/4 (S)
Comparators		TZP	>64/4 (R)
		CAZ	>16 (R)
		FEP	>16 (R)
		ATM	>16 (R)
		IPM	64 (R)
		MEM	256 (R)
		AMK	8 (S)
		GEN	>8 (R)
		CST	>1 (I)
		CIP	>2 (R)
		LVX	>4 (R)

^a^ C/T, ceftolozane/tazobactam; CZA, ceftazidime/avibactam; TZP, piperacillin/tazobactam; CAZ, ceftazidime; FEP, cefepime; ATM, aztreonam; IPM, imipenem; MEM, meropenem; AMK, amikacin; GEN, gentamicin; CST, colistin; CIP, ciprofloxacin; LVX, levofloxacin; MIC, minimum inhibitory concentration; S, susceptible; I, intermediate; R, resistant. ^b^ Antibiotic susceptibility (S, I, R) was interpreted according to the Clinical and Laboratory Standard Institute (CLSI) M100 guidelines. ^c^ MIC values for C/T, CZA, IPM, and MEM were determined using the standard broth microdilution method in accordance with the CLSI guidelines. MIC values for other agents were determined using the automated system (VITEK 2, bioMérieux).

## Data Availability

The original contributions presented in this study are included in the article. Further inquiries can be directed to the corresponding authors.

## Abbreviations

The following abbreviations are used in this manuscript:

MDR	Multidrug-resistance
CRPA	Carbapenem-resistant *Pseudomonas aeruginosa*
IDSA	Infectious Disease Society of America
WHO	World Health Organization
C/T	Ceftolozane/tazobactam
CZA	Ceftazidime/avibactam
PI	Prolonged infusion
CI	Continuous infusion
MIC	Minimum inhibitory concentration
KPC	*Klebsiella pneumonia* carbapenemase
NDM	New Delhi Metallo-β-lactamase
HFIM	Hollow fiber infection model
PBS	Phosphate-buffered saline
ATCC	American type culture collection
MHA	Mueller–Hinton agar
MHB	Mueller–Hinton broth
OD_600_	Optical density at 600 nm
CLSI	Clinical and Laboratory Standards Institute
CFU	Colony-forming unit
ECS	Extracapillary space
FDA	Food and Drug Administration
EMA	European Medicines Agency
MFDS	Ministry of Food and Drug Safety
*f*C_max,ss_	Free drug maximum concentration at steady state
*f*C_min,ss_	Free drug minimum concentration at steady state
*f*C_ss,avg_	Free drug steady-state average concentration
